# Why do patients attend out-of-hours GP services in Ireland?

**DOI:** 10.1007/s11845-025-04071-7

**Published:** 2025-08-19

**Authors:** Aisling Farrell, Alannah McCarthy, Roseanne Tobin, Elizabeth Bowen, Audrey Russell, Aisling Jennings

**Affiliations:** 1 Department of General Practice, University College Cork, Cork, Ireland; 2 Cork GP Training Scheme, University College Cork, Cork, Ireland; 3 Cork GP Training Scheme and Chairperson South West Doctors-On-Call Limited, Cork, Ireland

**Keywords:** Acute care, General practice, Out of hours

## Abstract

**Background:**

The attendance at out-of-hours GP services in Ireland has increased over the last number of years. However, the reasons for the increased demand have not been explored in the literature.

**Aims:**

The aim of this study was to identify the factors contributing to the use of out-of-hours GP services in Ireland from the patient’s perspective.

**Methods:**

A survey was designed and piloted prior to distribution. The survey was completed by patients attending an out-of-hours GP treatment centre in Cork, Ireland over a four-month period. Using a combination of forced choice items, free text boxes and five-point Likert scales, questions explored the reasons for attendance and experiences of patients with the out of hours service. Descriptive statistics were used to analyse the data on MS Excel.

**Results:**

80 people completed the survey. 75% (60/80) of participants stated that they had not attempted to contact their GP prior to contacting the out-of-hours centre. 49% (39/80) contacted the out-of-hours service as they felt that their issue was urgent. 36% (29/80) stated that they were unable to obtain an appointment with their GP and 15% (12/80) stated that the out-of-hours service was more convenient than regular GP hours. 29% (23/80) had symptoms for more than 4 days prior to contacting out-of-hours. The most common presentation was coryzal and upper respiratory tract symptoms, grouped collectively as ‘Ear Nose and Throat’ (ENT) symptoms at 34% (27/80).

**Conclusions:**

This study provides insight into the factors driving patient attendance at the out-of-hours GP service in Ireland. These factors include perceived urgency of symptoms, an inability to obtain an appointment with their own GP and in some cases, the convenience of out-of-hours services. There is a demand for targeted patient educational campaigns and increased resourcing for GP services during the daytime to reduce reliance on out-of-hours GP services.

## Introduction

General Practice (GP) co-operatives were first established in Ireland in 1998. The aim of these out-of-hours co-operatives was to reduce the onerous burden on GPs, particularly rural GPs, providing care to patients out of office hours while also maintaining busy daytime surgeries [1]. Approximately 93% of GPs in Ireland were part of an out-of hours co-operative as of 2017, which remains the most recent national estimate to the authors knowledge [2]. The attendance at out-of-hours GP services in Ireland has increased over the last number of years. In Ireland there are 13 GP out-of-hours co-operatives covering a total 92 out-of-hours GP treatment centres nationwide [3]. ‘Southdoc’ is one such GP out-of-hours co-operative in the south of Ireland. The increase in case numbers in this one GP out-of-hours co-operative demonstrates this increased demand. In 2021, there were a total of 203,095 cases seen in Southdoc, this rose to 211,975 in 2023. The delivery of out-of-hours care is a challenge, particularly due to rising demand and difficulties in recruiting GP to staff these services [4].

Similarly, international studies have demonstrated that GPs have experienced an increase in out-of-hours workload [5–7]. GP out-of-hours services are designed for urgent presentations. However, studies internationally have found that increasingly the GP out-of-hours service is being used to provide non-urgent care [8, 9].

There are a number of potential reasons why patients may choose to attend an out-of-hours GP service in Ireland. These may include an inability to attend their own GP, GP staffing shortages, use of service by patients with non-urgent issues and greater patient demands and expectations [10]. Population growth, GMS expansion and the impact of the covid pandemic are also likely contributors. However, the specific factors influencing patient decision-making in this context have not been well explored in the literature.

Urgent out-of-hours services are most effectively utilised when their purpose is understood and when service experiences align with patient expectations [11–13]. Understanding why patients choose to attend out-of-hours services is therefore critical to inform how these services are communicated, structured and delivered.

There is a lack of data in the Irish healthcare context pertaining to out-of-hours service use. This data is required to inform policy at a local and national level to allow practice changes and service reorganisation that could reduce unnecessary strain on the out-of-hours services.

The aim of this study was to identify the factors contributing to the use of out-of-hours GP services in Ireland from the patient’s perspective.

## Methods

This was a cross-sectional study of patient’s experiences with an out-of-hours GP service ‘Southdoc’ in the Republic of Ireland. This study focused on one treatment centre within this GP co-operative; the Cork city centre Southdoc service. As of 2022, there were 208 GPs affiliated with this out of hours treatment centre, catering for a population size of 224,004.

A survey tool, ‘Survey Monkey’ was designed to extract demographic data and information on the patient’s reason for attending the out-of-hours service and whether difficulty accessing their own GP influenced this attendance [14].

The survey was designed by the research team. It was piloted with patients from three GP Practices prior to distribution. Minor amendments were made to the survey as a result. The study was reviewed and approved by Medical Directors and the General Manager in South West Doctors-On-Call Ltd. Ethical approval was received from the Irish College of General Practitioner’s Research Ethics Committee. [ICGP_REC_2022_T07].

The study participants were patients, adults and children, attending an out-of-hours GP treatment centre in Cork, a city in the South of Ireland. Using existing attendance data, we estimated that approximately 4961 patients would attend this out-of-hours treatment centre during the 4 months of our study duration. We estimated the sample size required to adequately power this study to be 135 participants.

Participants were invited to participate via an opt-in QR code which was displayed on a poster in the patient waiting area in the treatment centre. Parents/guardians were invited to complete the survey on their child’s behalf. Following the scanning of the QR code, participants were brought to the online survey. The survey was held on Survey Monkey and was available for a four-month period from January to April 2023.

Using a combination of forced choice items, free text boxes and five-point Likert scales, questions explored experiences of patients with the out-of-hours service. The treating doctor was advised not to highlight the study to the patients attending the out-of-hours service as this may have put undue pressure on the patients to participate and affect the objectivity of the results. Descriptive statistics were used to analyse the data on MS Excel.

## Results

The survey data was collected between January 2023 and April 2023. 80 people responded.

Demographic data is presented in Table 1.

**Table 1:** Demographic of participants n (%)

Age	n (%)
< 6 years old	24 (30%)
7–17 years old	20 (25%)
18–64 years old	35 (44%)
> 65 years old	1 (1%)
Sex	
Male	33 (41%)
Female	46 (58%)
Rather not say	1 (1%)
Medical card holder	
Yes	40 (50%)
No	39 (49%)
Rather not say	1 (1%)

### Out-of-hours use

All patients were registered with a GP. 75% (*n* = 60) of participants stated that they had not attempted to contact their GP prior to contacting the out-of-hours centre.

**Fig. 1:**
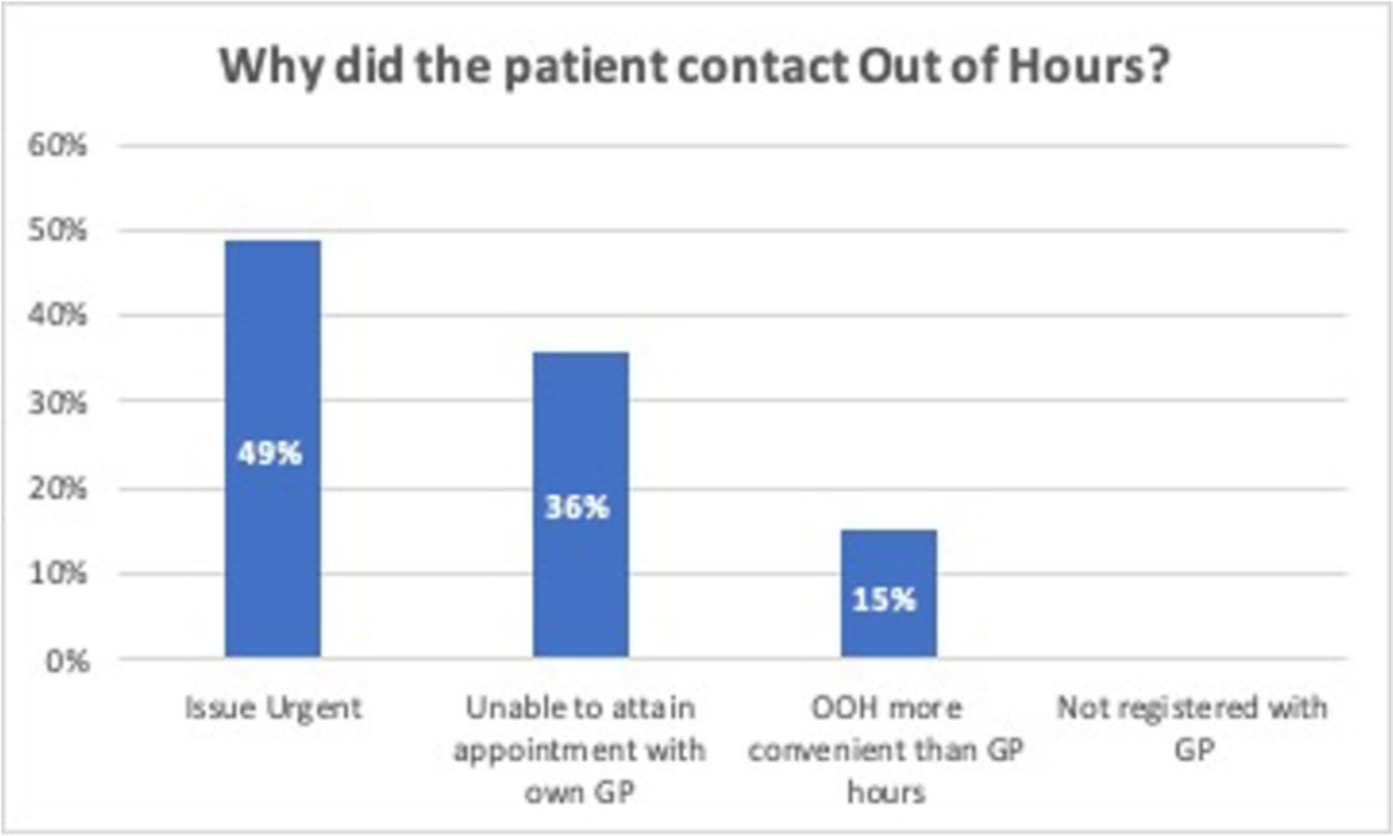
Patients’ reasons for choosing the out-of-hours service

The reasons patients gave for contacting the out-of-hours centre varied, see Fig. 1. Almost half of patients (49%, *n* = 39) contacted the out-of-hours service as they felt that their issue was urgent. 36% (*n* = 29) stated that they were unable to obtain an appointment with their GP and 15% (*n* = 12) stated that the out-of-hours service was more convenient than regular GP hours.

Patients were also asked how often they frequented the out-of-hours service in the past year. Most participants (86%, *n* = 68) stated that they had visited under 5 times. 14% (*n* = 11) stated they had attended out-of-hours between 5 and 10 times in the past year. No one had attended the out-of-hours service more than 10 times in the past year.

### Symptom duration

**Fig. 2:**
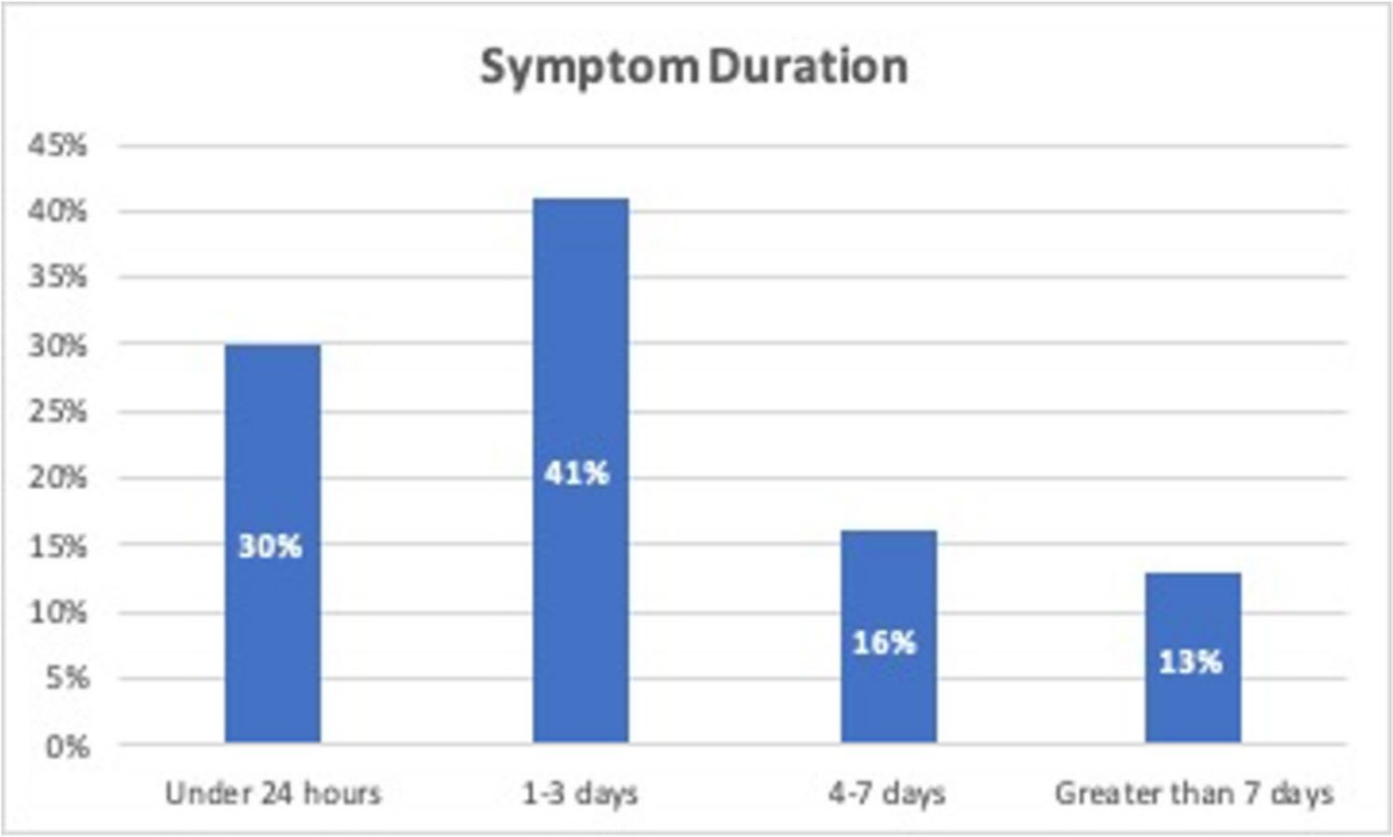
Symptom duration

30% (*n* = 24) reported their symptoms had started in the preceding 24 h, most patients (41%, *n* = 33) had symptoms for 1–3 days and the remaining 16% (*n* = 13) had symptoms between 4–7 days. 13% (*n* = 10) of patients reported that they had symptoms for over a week before they contacted the out-of-hours service, see Fig. 2.

**Fig. 3:**
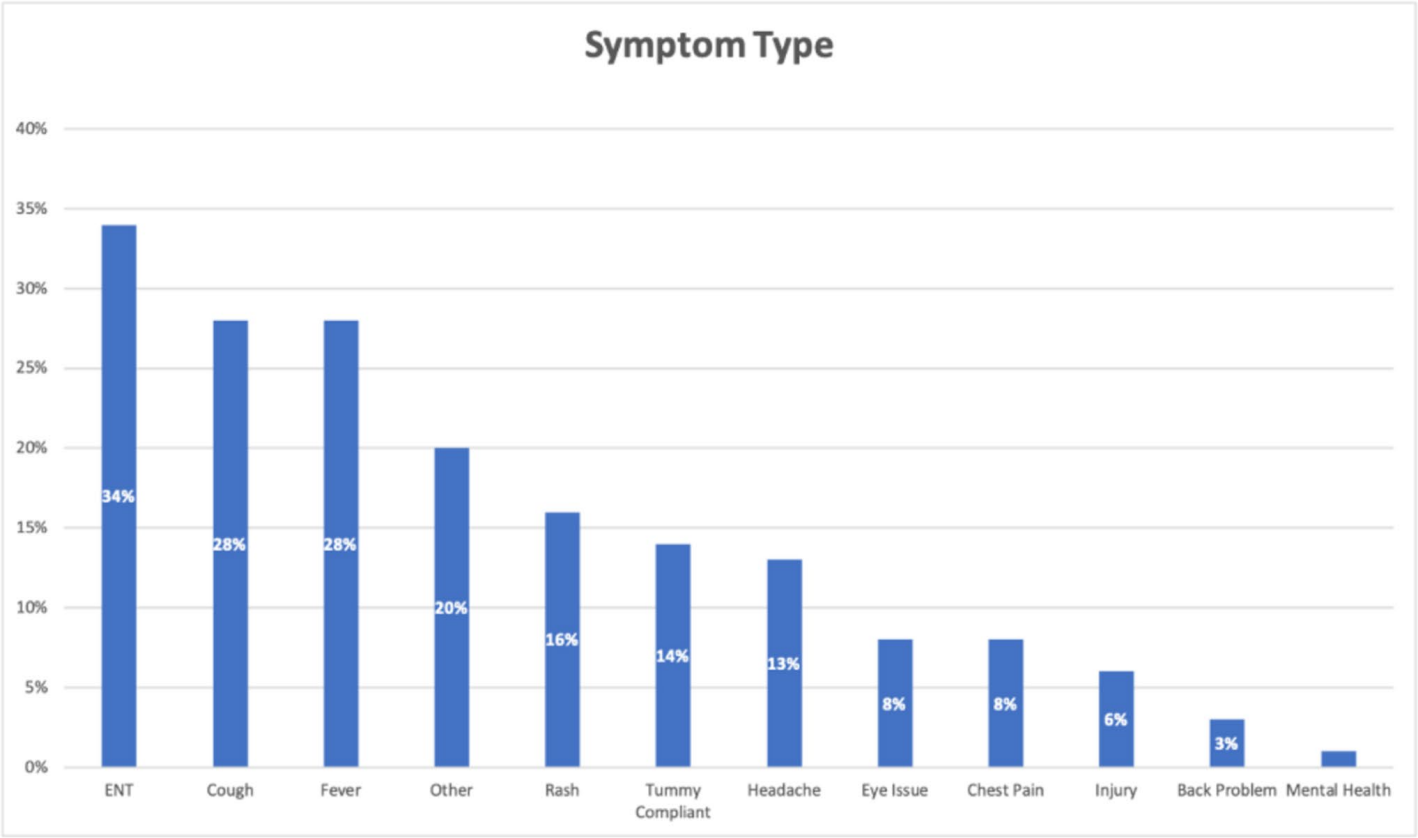
Symptom type

The symptoms that patients presented to the out-of-hours service with were varied. Some patients described multiple symptoms. The most common symptoms were ear, nose and throat related (coryzal symptoms, sore throat, sore ears) and grouped collectively as Ear Nose and Throat (ENT) (34%, *n* = 27) followed by cough (28%, *n* = 22) and fever (28%, *n* = 22). Other symptoms included rash, chest pain and injury, see Fig. 3.

### Outcome

92% (*n* = 74) were managed exclusively in the GP out-of-hours service, with 8% (*n* = 6) referred to hospital. 68% (*n* = 54) of patients were issued with a prescription. 6% (*n* = 5) were advised to follow up with their GP. 19% (*n* = 15) had other outcomes, which were not specified.

### Patient satisfaction

81% (*n* = 65) of patients were either very satisfied or satisfied with their encounter with the out-of-hours centre. 11% (*n* = 9) of patients however were dissatisfied or very dissatisfied. Reasons for this dissatisfaction were provided in the free text comments and were largely related to the lengthy wait time for a call-back and wait time for a doctor’s appointment.

## Discussion

The primary aim of this study was to assess the underlying factors contributing to patient attendance in the out-of-hours GP services in Ireland. The findings from this study demonstrate that the majority of patients, 75%, did not contact their own GP prior to attending the out-of-hours service. This suggests that one of the reasons that patients elect to attend the out-of-hours services may be a perceived barrier in accessing an appointment with their own GP.

The fact that 29% of patients had symptoms for greater than 4 days suggests that a number of these visits may not have been clinically urgent. From this we can infer that use of out of hours services may not always be in line with the aim of the out-of-hours service which is to see urgent issues.

8% of all cases seen during this period were referred to hospital, demonstrating that 92% of all cases are managed in the community setting, similar to international findings [15].

Overall, patient satisfaction with the out-of-hours service was high. Factors including perceived urgency of symptoms, an inability to obtain an appointment with their own GP and in some cases, the relative convenience of out-of-hours services highlight the need for targeted interventions to improve GP accessibility.

### Strengths and limitations

This study has a number of strengths. It provides a valuable insight into the Irish healthcare system’s specific challenges, contributing to the relatively sparse literature on out-of-hours service use in Ireland. The focus on patient satisfaction with the service also provides actionable feedback for healthcare providers and policymakers.

However, there were some limitations. The response rate was lower than anticipated with 80 responders. This study relied on voluntary participation by patients themselves and they had to take the initiative to do so. This is a recognised limitation as younger patients are likely more accustomed to using QR codes to access online material. An average of 14.8% of people attending ‘Southdoc’ from 2021–2023 were aged 70 years and over. As only 1% of responders in this study were over 65 years old, this suggests a response bias and unlikely to be a representative number for patients attending out-of-hours. This unexpectedly low figure may have been influenced by the study design as participants required a smart phone to participate and thus may have excluded older attendees.

The study questionnaire also had some limitations. Symptoms were obtained but discharge diagnosis was not identified. Also this study identified that a prescription was required but not the content of same.

The study pertained to one out-of-hours treatment centre in the South of Ireland which may limit the study’s external validity as it is confined to a certain geographical area.

### Implications

The findings of this study have significant implications for healthcare delivery and the management of out-of-hours GP services in Ireland. The most striking result is that 75% of participants did not attempt to contact their regular GP before using out-of-hours services. There are a number of potential reasons for this. Firstly, it could demonstrate that the patients became acutely unwell outside of daytime surgery hours. It could also represent an inappropriate use of the out-of-hours services, in that patients did not make any attempt to contact their own GP but rather contacted the out-of-hours services in the first instance.

Another possibility is that these patients may have had difficulty accessing their own GP, which is a recognised consequence of the increase demands and longer wait times for appointments in General Practice [16]. This aligns with international findings, where barriers to accessing regular daytime GP care, such as long waiting times, push patients toward out-of-hours services and emergency department even for non-urgent issues [16–18]. Increasing the capacity in daytime GP services for our growing population will have a role in reducing reliance on out-of-hours GP services going forward.

Of note, 25% of patients had contacted their own GP and still required out-of-hours services. Further research into this group would be worthwhile to ascertain the reasons why some patients did not contact their GP initially and for those who did, to establish the reasons why they then contacted the out-of-hours services.

The most common symptom encountered was ENT issues (34%), followed by cough and fever (28% each). This is in keeping with a large Australian study assessing out-of-hours GP attendances [19]. This may also have been influenced by the seasonality of such complaints, as our study was conducted from January to April 2023. 29% of participants had symptoms for greater than 4 days. These results demonstrate that use of out-of-hours services may not always be appropriate. Similar findings have also been noted internationally, where perceived urgency of complaints increases attendance at out-of-hours services [19–21]. This highlights an area where patient self-care resources, for example ‘undertheweather.ie^’^ and educational campaigns. on appropriate use of out-of-hours GP services and self-care may have utility in reducing strain on an already busy out-of-hours services [22].

Most patients were pleased with the service they received, but some patient expressed frustration with delays. The reasons cited by the patients who were dissatisfied surrounded delay in call back time and delay in seeing a doctor.

Future research should ensure accessibility to patients who may be ruled out by reliance on certain technology. Additionally, including a larger sample size across multiple geographical areas would strengthen the reliability of the findings at a national level. Qualitative research to explore patient factors in more depth, as well as research examining the role of online GP services, attendance at local injury units or private express care facilities in out-of-hour GP attendance is required.

## Conclusion

This study provides insight into the factors driving patient attendance at the out-of-hours GP service in Ireland. Increased resourcing and additional capacity within day time GP services for our growing population will have a role in reducing reliance on out-of-hours GP services. Addressing these challenges will not only improve patient outcomes but also alleviate pressure on the out-of-hours services, ensuring they remain available for those who will benefit most from them. In doing so, we can create a more efficient, sustainable and patient-focused healthcare system in Ireland. Thank you to Southdoc management, administrative staff and the medical director for supporting this research.

## Data Availability

The data that support the findings of this study are available from the corresponding author, [AF], upon reasonable request.

## Declarations

This is a statement of compliance with ethical standards. This is to confirm the study protocol was reviewed and approved by the Irish College of General Practitioner’s Research Ethics Committee. AJ is in receipt of a Career Development Grant from the Irish College of General Practitioners (R17971).
